# Parental Adverse Childhood Experiences and Perpetration of Child Physical Punishment in Wales

**DOI:** 10.3390/ijerph191912702

**Published:** 2022-10-04

**Authors:** Karen Hughes, Kat Ford, Mark A. Bellis, Rebekah Amos

**Affiliations:** 1 World Health Organization Collaborating Centre on Investment for Health and Well-Being, Public Health Wales, Wrexham LL13 7YP, UK; 2 College of Human Sciences, Bangor University, Wrexham LL13 7YP, UK

**Keywords:** adverse childhood experiences, violence, physical punishment, parents, children

## Abstract

Child physical punishment is harmful to children and, as such, is being prohibited by a growing number of countries, including Wales. Parents’ own childhood histories may affect their risks of using child physical punishment. We conducted a national cross-sectional survey of Welsh adults and measured relationships between the number of adverse childhood experiences (ACEs) parents (n = 720 with children aged < 18) had suffered during childhood and their use of physical punishment towards children. Overall, 28.2% of parents reported having ever physically punished a child, and 5.8% reported having done so recently (in the last year). Child physical punishment use increased with the number of ACEs parents reported. Parents with 4+ ACEs were almost three times more likely to have ever physically punished a child and eleven times more likely to have done so recently (vs. those with 0 ACEs). The majority (88.1%) of parents that reported recent child physical punishment had a personal history of ACEs, while over half reported recently having been hit themselves by a child. Child physical punishment is strongly associated with parents’ own ACE exposure and can occur within the context of broader conflict. Prohibiting physical punishment can protect children and, with appropriate family support, may help break intergenerational cycles of violence.

## 1. Introduction

The physical punishment of children is recognised internationally as a form of violence that harms children and violates their rights to dignity and protection [1]. Child physical punishment has been shown to be a poor method of regulating child behaviour and is linked to worsening child behaviour rather than its improvement [2]. Such punishment can increase children’s risks of externalizing behaviours such as aggression; internalizing issues such as mental ill health; and involvement in other forms of violence such as intimate partner violence later in life [2,3,4]. The United Nations has called on states to prohibit child physical punishment [1] and has included a target to end all forms of violence against children in the Sustainable Development Goals (target 16.2) [5]. Globally, by 2022, 63 states had prohibited child physical punishment in all settings, accounting for just 14% of the world’s children [6]. Evidence shows that such prohibitions can reduce support for, and prevalence of, child physical punishment [2]. However, even in countries where prohibitions have been successfully implemented and social norms favour non-violent child discipline, a proportion of parents can continue to physically punish their children [7].

A range of individual, relational, cultural and societal factors can affect the risk of parents physically punishing children, including their own experience of abusive parenting and other childhood adversities [8,9]. Adverse childhood experiences (ACEs) such as child maltreatment, witnessing domestic violence and caregiver substance misuse, show cumulative relationships with poor health and social outcomes in later life, including health-harming behaviours such as alcohol and drug use; mental illness; and involvement in various forms of violence [10]. Accordingly, parental ACEs have been associated with multiple risk factors for child maltreatment, perpetration of child maltreatment, and lower protective factors such as resiliency and social connections [11,12]. Despite this, few studies have explored relationships between parents’ history of ACEs and their use of physical punishment towards children. However, a recent study explored relationships between parents’ exposure to individual ACEs and child spanking, finding that children whose parents had suffered physical abuse, emotional abuse, spanking, and household mental illness during childhood had increased likelihood of having been spanked [13].

Understanding factors associated with parental use of child physical punishment is essential to inform interventions to prevent such punishment and help break intergenerational cycles of violence. In 2022, Wales joined the growing number of countries to ban physical punishment of children in all settings. Using data collected a year prior to legislative change, this study explores relationships between Welsh parents’ exposure to cumulative ACEs whilst growing up and their use of physical punishment towards children.

## 2. Materials and Methods

A national survey of Welsh adults (age ≥ 18) was undertaken between December 2020 and March 2021 (during national COVID-19 lockdown restrictions) to measure relationships between ACEs, health and behaviour [14]. The survey was conducted by telephone (n = 2326) using a sample stratified by age, Welsh Health Board and residential deprivation (using the Welsh Index of Multiple Deprivation [WIMD] [15]). Due to difficulty accessing younger adults via telephone, an online survey was also disseminated via a commercial panel. The online survey (n = 650) included all adult age groups, targeted proportionate to population demographics. Sampling and data collection were undertaken by a professional market research company. Potential participants were informed of the nature of the survey, including its voluntary, anonymous and confidential nature, and informed consent was recorded using opt-in consent. For the telephone survey, contact was made with 6763 individuals, of whom 98 did not meet the inclusion criteria, 4062 declined to participate and the remainder (n = 2603) agreed. Of those agreeing to participate, 277 did not fall within the required age quota of their area. Thus, the participation rate for the telephone sample for eligible participants meeting the quota sampling was 36.4% (2326/6388). It was not possible to calculate a participation rate for the online sample. For analysis, the sample was limited to parents with children aged < 18 (n = 735). Fifteen cases were excluded due to missing key data (gender, age group, ACEs or child physical punishment) for a final sample of 720 parents.

The questionnaire measured participants’ exposure to nine ACE types before the age of 18 using the Centers for Disease Control and Prevention short ACE tool [16]: (1) physical, (2) verbal and (3) sexual abuse; (4) parental separation; (5) exposure to domestic violence; and household member (6) mental illness, (7) alcohol abuse, (8) drug abuse and (9) incarceration. Consistent with other studies [10], and to ensure adequate sample sizes for analysis, the number of ACEs participants reported was summed for analysis (0 ACEs, 1 ACE, 2–3 ACEs, 4+ ACEs). Child physical punishment was measured by two questions asking: how many times participants had ever smacked or slapped a child for misbehaving (coded to never/at least once for analysis) and whether they had smacked or slapped a child to punish them for their behaviour in the last 12 months (no/yes). Participants were also asked if, in the last 12 months, they had: hit another adult for any reason, including to defend themselves; been hit by an adult; and been hit by a child (aged < 18 years). Demographic variables included age, gender, ethnicity and residential deprivation quintile (coded from postcode using the WIMD).

Statistical analysis was undertaken in SPSS v24 (Armonk, NY, USA) and used chi squared for bivariate analysis and logistic regression for multivariate analysis.

## 3. Results

Participant demographics, ACE exposure levels and associations with child physical punishment are shown in Table 1. Over a quarter (28.2%) of parents reported having ever physically punished a child and 5.8% reported having done so recently (last 12 months). There were no associations between child physical punishment and parental gender or ethnicity. Ever physically punishing a child varied by deprivation and was reported more by older parents, but there was no association between recent child physical punishment and parental age or deprivation. ACE count was significantly associated with both physical punishment measures. Having ever physically punished a child increased from 21.6% in those with no ACEs to 36.0% in those with 4+ ACEs, while recent physical punishment increased from 1.7% to 17.5%, respectively. In multivariate analysis (Table 1), adjusted odds of child physical punishment increased with ACE count. Parents with 4+ ACEs were almost three times more likely to have ever physically punished a child and over eleven times more likely to have done so recently.

Whilst the number of parents reporting recently physically punishing a child was small, the majority of these parents reported having suffered at least one ACE themselves in childhood (88.1% vs. 57.8% of parents that had not recently physically punished a child) and almost half (47.6% vs. 13.9%) reported 4+ ACEs. The most common ACE types reported were parental separation (52.4% vs. 32.8% of parents that had not recently physically punished a child), physical abuse (47.5% vs. 18.0%), domestic violence (46.3% vs. 18.7%) and emotional abuse (45.2% vs. 26.3%). In the past year, 57.1% of parents that reported recently inflicting physical punishment also reported having been hit by a child themselves (vs. 12.1% of other parents), while 14.3% reported having been hit by an adult (vs. 5.5%) and 16.7% having hit another adult (vs. 3.2%).

## 4. Discussion

Our study, conducted a year prior to the prohibition of child physical punishment in Wales, found that only a minority of Welsh parents reported having used physical punishment towards children in the previous year. However, the risks of using physical punishment were strongly associated with parents’ own exposure to ACEs in childhood. Odds of parents reporting use of physical punishment increased with ACE count, with those that reported 4+ ACEs being over 11 times more likely to report the use of physical punishment in the last year. In fact, the vast majority of parents that reported recent use of physical punishment had suffered at least one ACE themselves in childhood, often including violence.

Our findings are consistent with studies elsewhere identifying intergenerational transmission of violence and other ACEs [17]. Existing evidence suggests various pathways through which such intergenerational transmission can occur. These include the harmful and lasting impacts of toxic stress imposed by ACEs on children’s neurological development and stress response, which can affect emotional regulation, social functioning, health-risk behaviours and mental health [18], all of which may impact on parenting behaviours in later life. Further, exposure to violence in the home during childhood may lead individuals to view violence as normal behaviour and an acceptable way of resolving conflict and disciplining a child [17]. In our study, parents who used physical punishment towards children often also reported being hit by children themselves, further reflecting intergenerational transmission. They also reported involvement in violence with other adults more than parents that did not use physical punishment, suggesting that child physical punishment can occur in a context of broader family conflict.

These findings have implications for the prevention of physical punishment against children, and the successful implementation of legislation to prohibit child physical punishment. Importantly, in our study most parents that reported ACEs did not report the use of child physical punishment. However, it is critical that both policymakers and professionals who work with parents and children understand how parents’ own childhood experiences may affect their parenting skills, as well as their vulnerability to other forms of violence and risk factors such as mental ill health. This understanding can support the development and targeting of appropriate messaging and parenting support. There are a range of effective parenting programmes that can reduce the risk of violence towards children [19] and a growing evidence base for programmes tailored to support parents with a history of ACEs, often focusing on attachment, relationships and emotional regulation [17]. Sensitive enquiry about parents’ personal childhood experiences [17] in maternity or early-years services can provide health professionals with the opportunity to identify vulnerability and provide support, including reflection, discussion, education and referral to services where appropriate.

Our study has several limitations. In line with other retrospective cross-sectional studies, it relied on parents’ recall and willingness to report sensitive issues, including ACEs, child physical punishment and involvement in other violence. The study was conducted during a period of national COVID-19 lockdown restrictions that included stay at home orders, social distancing and the closure of non-essential services. Such restrictions have been linked to elevated levels of parenting stress, worsening mental ill health and increased reports of child abuse and neglect [20]. Despite this, levels of recent child physical punishment reported by parents were low. The timing of the study a year prior to implementation of national prohibition on child physical punishment may have impacted parents’ willingness to report this behaviour. However, findings also suggest that social norms in Wales were already supportive of non-violent child punishment prior to legislative change. Thus, specific risk factors such as ACEs may help identify parents that are at risk of continuing to use physical punishment towards children. However, attitudes towards, and use of, child physical punishment vary widely across countries [7], and consequently our findings cannot be generalised internationally. In countries where child physical punishment remains normative and socially acceptable, the successful implementation of such legislation would require a package of measures to change social norms, educate parents and develop parenting skills more broadly [21].

## 5. Conclusions

Exposure to ACEs during childhood can impact individuals’ parenting behaviours later in life, with parents that have suffered ACEs being at increased risk of using physical punishment towards children. Action to prevent child physical punishment should recognise the additional challenges faced by parents that have suffered ACEs and tailor programmes accordingly. While banning physical punishment can be an essential step in protecting children from this form of violence, it should be combined with appropriate support for families to break intergenerational cycles of violence.

## Figures and Tables

**Table 1:** Participant characteristics and bivariate and multivariate associations with child physical punishment.

Sample		n (%)	Physically Punished a Child							
			Ever				In the Last Year			
			%	*p*	AOR (95%CIs)	*p*	%	*p*	AOR (95%CIs)	*p*
All		720 (100.0)	28.2				5.8			
Gender	Male	240 (33.3)	30.4		Ref		5.0		Ref	
	Female	480 (66.7)	27.1	0.349	1.14 (0.78–1.65)	0.500	6.3	0.500	1.25 (0.59–2.61)	0.559
Age	18–29	69 (9.6)	17.4		Ref	<0.001	7.2		Ref	0.469
	30–39	231 (32.1)	19.5		1.27 (0.62–2.60)	0.521	8.7		1.48 (0.52–4.26)	0.465
	40–49	271 (37.6)	31.4		2.70 (1.34–5.45)	0.006	3.7		0.77 (0.24–2.45)	0.658
	50+	149 (20.7)	40.9	<0.001	4.15 (1.97–8.75)	<0.001	4.7	0.101	1.14 (0.32–4.05)	0.836
Deprivation quintile	(Most) 1	141 (19.6)	22.0		Ref	0.139	6.4		Ref	0.921
	2	135 (18.8)	33.3		1.67 (0.95–2.91)	0.073	7.4		1.43 (0.54–3.81)	0.469
	3	146 (20.3)	21.9		0.99 (0.56–1.78)	0.986	6.2		1.33 (0.49–3.62)	0.577
	4	151 (21.0)	34.4		1.70 (0.97–2.97)	0.063	5.3		1.49 (0.52–4.26)	0.456
	(Least) 5	147 (20.4)	29.3	0.036	1.31 (0.75–2.32)	0.345	4.1	0.806	1.06 (0.35–3.24)	0.918
BAME *	No	687 (95.4)	27.9		Ref		5.8		Ref	
	Yes	33 (4.6)	33.3	0.502	1.34 (0.60–2.96)	0.483	6.1	0.955	0.93 (0.20–4.22)	0.923
ACE count	0	291 (40.4)	21.6		Ref	<0.001	1.7		Ref	<0.001
	1	155 (21.5)	29.0		1.62 (1.02–2.56)	0.041	4.5		2.50 (0.78–8.08)	0.125
	2–3	160 (22.2)	33.8		2.29 (1.45–3.61)	<0.001	6.3		3.70 (1.22–11.17)	0.020
	4+	114 (15.8)	36.0	0.007	2.89 (1.72–4.80)	<0.001	17.5	<0.001	11.74 (4.16–33.18)	<0.001

* BAME = Black, Asian and minority ethnic group. ACE = adverse childhood experience. AOR = adjusted odds ratio. Ref = reference category.

## Data Availability

The data presented in this study are available on request from the corresponding author.

## References

[1] (2006). UN Committee on the Rights of the Child: Geneva, Switzerland. https://digitallibrary.un.org/record/583961.

[2] United Nations Sustainable Development Goals. https://www.unodc.org/roseap/en/sustainable-development-goals.html.

[3] Heilmann A., Mehay A., Watt R.G., Kelly Y., Durrant J.E., van Turnhout J., Gershoff E.T. (2021). Physical punishment and child outcomes: A narrative review of prospective studies. Lancet.

[4] Afifi T.O., Mota N., Sareen J., MacMillan J.L. (2017). The relationships between harsh physical punishment and child maltreatment in childhood and intimate partner violence in adulthood. BMC Public Health.

[5] Gershoff E.T., Grogan-Kaylor A. (2016). Spanking and child outcomes: Old controversies and new meta-analyses. J. Fam. Psychol..

[6] End Corporal Punishment. https://endcorporalpunishment.org/countdown/.

[7] Zolotor A.J., Puzia M.E. (2010). Bans against corporal punishment: A systematic review of the laws, changes in attitudes and behaviours. Child Abus. Rev..

[8] Langevin R., Marshall C., Kingsland E. (2021). Intergenerational cycles of maltreatment: A scoping review of psychosocial risk and protective factors. Trauma Violence Abus..

[9] Hughes M., Cossar J. (2016). The relationship between maternal childhood emotional abuse/neglect and parenting outcomes: A systematic review. Child Abus. Rev..

[10] Hughes K., Bellis M.A., Hardcastle K.A., Sethi D., Butchart A., Mikton C., Jones L., Dunne M.P. (2017). The impact of multiple adverse childhood experiences on health: A systematic review and meta-analysis. Lancet Public Health.

[11] Buffarini R., Hammerton G., Coll C.V.N., Cruz S., Freitas da Silveira M., Murray J. (2022). Maternal adverse childhood experiences (ACEs) and their associations with intimate partner violence and child maltreatment: Results from a Brazilian birth cohort. Prev. Med..

[12] Steele H., Bate J., Steele M., Dube S.R., Danskin K., Knafo H., Nikitiades A., Bonuck K., Meissner P., Murphy A. (2016). Adverse childhood experiences, poverty, and parenting stress. Can. J. Behav. Sci..

[13] Afifi T.O., Salmon S., Stewart-Tufescu A., Taillieu T. (2022). An examination of parents’ adverse childhood experiences (ACEs) history and reported spanking of their child: Informing child maltreatment prevention efforts. Int. J. Environ. Res. Public Health.

[14] Bellis M.A., Hughes K., Ford K., Madden H.C.E., Glendinning F., Wood S. (2022). Associations between adverse childhood experiences, attitudes towards COVID-19 restrictions and vaccine hesitancy: A cross-sectional study. BMJ Open.

[15] Welsh Government (2019). Welsh Index of Multiple Deprivation. https://gov.wales/welsh-index-multiple-deprivation-full-index-update-ranks-2019.

[16] Centers for Disease Control and Prevention Behavioural Risk Factor Surveillance System Adverse Childhood Experience (ACE) Module. https://www.cdc.gov/violenceprevention/acestudy/pdf/BRFSS_Adverse_Module.pdf.

[17] Narayan A.J., Lieberman A.F., Masten A.S. (2021). Intergenerational transmission and prevention of adverse childhood experiences (ACEs). Clin. Psychol. Rev..

[18] Berens A.E., Jensen S.K.G., Nelson C.A. (2017). Biological embedding of childhood adversity: From physiological mechanisms to clinical implications. BMC Med..

[19] Desai C.C., Reece J.-A., Shakespeare-Pellington S. (2017). The prevention of violence in childhood through parenting programmes: A global review. Psychol. Health Med..

[20] Calvano C., Engelke L., Di Bella J., Kindermann J., Renneberg B., Winter S.M. (2022). Families in the COVID-19 pandemic: Parental stress, parent mental health and the occurrence of adverse childhood experiences—Results of a representative survey in Germany. Eur. Child Adolesc. Psychiatry.

[21] Global Partnership to End Violence against Children (2021). Laying the Foundations for Non-Violent Childhoods: Putting Prohibition of Corporal Punishment of Children into Practice: Implementation Guidance.

